# Alarm Pheromone Composition and Behavioral Activity in Fungus-Growing Ants

**DOI:** 10.1007/s10886-017-0821-4

**Published:** 2017-02-28

**Authors:** Victoria C. Norman, Thomas Butterfield, Falko Drijfhout, Kiah Tasman, William O. H. Hughes

**Affiliations:** 1 School of Life Sciences, University of Sussex, Brighton, East Sussex, BN1 9QG UK; 2 Chemical Sciences Research Centre, Keele University, Staffordshire, UK

**Keywords:** Leaf-cutting ant, Mandibular gland, Caste, Attini, Division of labor, Alarm pheromone

## Abstract

Chemical communication is a dominant method of communication throughout the animal kingdom and can be especially important in group-living animals in which communicating threats, either from predation or other dangers, can have large impacts on group survival. Social insects, in particular, have evolved a number of pheromonal compounds specifically to signal alarm. There is predicted to be little selection for interspecific variation in alarm cues because individuals may benefit from recognizing interspecific as well as conspecific cues and, consequently, alarm cues are not normally thought to be used for species or nestmate recognition. Here, we examine the composition of the alarm pheromones of seven species of fungus-growing ants (Attini), including both basal and derived species and examine the behavioral responses to alarm pheromone of *Acromyrmex* leaf-cutting ants, the sister genus to the highly studied *Atta* leaf-cutting ants. We find surprisingly high interspecific variation in alarm pheromone composition across the attine phylogeny. Interestingly, the active component of the alarm pheromone was different between the two leaf-cutting ant genera. Furthermore, in contrast to previous studies on *Atta*, we found no differences among morphological castes in their responses to alarm pheromone in *Acromyrmex* but we did find differences in responses among putative age classes. The results suggest that the evolution of alarm communication and signaling within social insect clades can be unexpectedly complex and that further work is warranted to understand whether the evolution of different alarm pheromone compounds is adaptive.

**Electronic supplementary material:**

The online version of this article (doi:10.1007/s10886-017-0821-4) contains supplementary material, which is available to authorized users.

## Introduction

Alarm communication is shown by a wide variety of organisms, including vertebrates, invertebrates and even plants (Chivers and Smith 1998; Shah 2009; Wyatt 2014). Alarm communication is particularly beneficial when individuals live in groups, because the rapid communication of threats to group members enables groups to form collective responses to stimuli (Nault and Phelan 1984; Verheggen et al. 2010). In many organisms, these alarm signals are visual (Murphy 2006; le Roux et al. 2008), but in insects they are predominantly chemical, with alarm pheromones being the secondly most commonly produced class of compounds among this group of organisms (Regnier and Law 1968; Verheggen et al. 2010).

Alarm communication is particularly diverse in the eusocial insects such as ants, in which a wide variety of compounds function as stimuli for alarm behaviors (Bortolotti and Costa 2014; Cammaerts et al. 1983; Crewe et al. 1972). These behaviors allow individuals within a colony to respond rapidly and appropriately to an alarm stimulus. There are two main behavioral responses to alarm cues, each of which serve separate functions (Verheggen et al. 2010; Wilson and Regnier 1971). The first is a panic response, in which responders show escape or flight behaviors to disperse from the threat. The second is an aggressive response, in which workers are attracted to, and attack, the threat. These responses can depend upon a variety of factors, including concentration of pheromone, compounds within the pheromone, colony size, and the spatial context of the communication (Hölldobler and Wilson 1990; Hughes and Goulson 2001; Kerr et al. 1973; Vander Meer and Alonso 1998).

We may also expect differences among individual workers in their response to alarm cues. Polyethism in complex eusocial insect societies can be based on age (temporal polyethism) or morphological phenotype (caste). Many insects show a specialized defensive caste, be it guards in honey bee colonies, or morphologically specialized soldiers in stingless bee, ant, termite, aphid or thrips colonies (Grüter et al. 2012; Hölldobler and Wilson 2010a; Shibao 1998). These individuals are often the first line of defence for the group and respond more aggressively to defensive stimuli (Whitehouse and Jaffe 1996). There is variation among colonies in alarm responses as well as between species; for example, as in the greater defensiveness of Africanized honey bees compared to their European counterparts (Collins et al. 1982; Giray et al. 2000). However, the reduced need for specificity in alarm pheromones compared to other pheromones means that there is predicted to be relatively little selection for the evolution of species-specific alarm pheromones (Blum 1969; Regnier and Law 1968; Vander Meer and Alonso 1998). Indeed, in other organisms, such as fish or crustaceans, a lack of species specificity can be an advantage in alarm cues (Commens and Mathis 1999; Laforsch et al. 2006).

The tribe Attini provides an ideal system for testing this hypothesis within social insects. This clade exhibits varying levels of social complexity. Leaf-cutting ants (*Atta* and *Acromyrmex*) are the most derived clade within the fungus-growing ants (tribe Attini), all of which are characterized by culturing a mutualistic fungal crop. The two ecologically dominant leaf-cutting ant genera are distinguished from other attines by cultivating their fungal crop, generally, on fresh vegetation and having much larger and more complex societies, with thousands to millions of polymorphic workers compared to tens to hundreds of monomorphic workers in the basal attines (Weber 1972). The higher leaf-cutting genera of *Acromyrmex* and *Atta* show some of the most complex forms of division of labor with extreme caste polymorphism, in which workers can vary in size by as much as six-fold ( Hölldobler and Wilson 2010b; Wilson 1980). Within leaf-cutting ant colonies, smaller workers tend to carry out internal work within the nest, such as caring for the brood and fungus garden, larger workers tend to carry out foraging and nest defence, while soldiers (only present in *Atta*) specialize normally in nest defence (Wetterer 1995; Wilson 1980). As with many other ant and bee species, the alarm pheromone in leaf-cutting ants is produced by the mandibular glands and is released when the mandibles are gaped in response to an alarm stimulus (Blum 1969; Verheggen et al. 2010). The compositions of the alarm pheromones in *Atta* have been relatively well studied, and all six of the species so far investigated (*A. bisphaerica*, *A. capiguara*, *A. laevigata*, *A. opaciceps* and *A. sexdens*) have 4-methyl-3-heptanone as the predominant and most active component (Blum et al. 1968; Crewe and Blum 1972; Do Nascimento et al. 1993; Francelino et al. 2006; Hernandez et al. 1999; Hughes et al. 2001a; Moser et al. 1968;). Depending on the species and caste, the alarm pheromone of *Atta* can also contain other volatile compounds, with as many as 41 volatile compounds found in head extracts (Hughes et al. 2001a), but the roles of these other compounds are unclear. There is also clear evidence of polymorphism in the composition, activity and receptiveness of *Atta* ants to alarm pheromone. While large workers and particularly soldiers tend to produce relatively complex pheromonal mixtures, the alarm pheromone of small workers (minims) in *Atta* is simpler, consisting predominantly of 4-methyl-3-heptanone (Do Nascimento et al. 1993; Hernandez et al. 1999; Hughes et al. 2001a). This suggests that the small workers in *Atta* may produce a particularly potent alarm pheromone in terms of composition, as the quantities they produce are lower, due to their smaller mandibular glands, than those of their larger nestmates (Hernández and Caetano 1995). Although all castes respond to alarm pheromone (Wilson 1980), field studies have found that smaller workers, although less abundant on foraging trails, are disproportionately abundant in the ants responding to alarm stimuli, suggesting that they may play a key role in detecting and responding to threats (Hughes and Goulson 2001).

The rich literature on the composition and behavioral activity of alarm pheromones in *Atta* leaf-cutting ants contrasts markedly with the very limited investigation of alarm pheromones in other fungus-growing ants. Only two studies have investigated the chemical composition of the mandibular gland pheromones in other Attini, and these suggest intergeneric differences, with eight *Trachymyrmex* species appearing to lack 4-methyl-3-heptanone (Adams et al. 2012; Crewe and Blum 1972). These results are intriguing because they suggest that different fungus-growing ant taxa may have evolved different alarm pheromone compounds, in contrast to the predicted lack of selection for interspecific variation in alarm pheromone. However, detailed analyses of the composition of alarm pheromones, and controlled assays of behavioral activity, are needed for *Acromyrmex* and other attines in order to resolve this. Here, we identify and compare the chemical composition of the alarm pheromones of two species of *Atta* that have not previously been investigated (*At. colombica* and *At. cephalotes*), two species of *Acromyrmex* (*Ac. echinatior* and *Ac. octospinosus*), two other ‘higher’ attines (*Sericomyrmex amabilis* and *Trachymyrmex cornetzi*), and the ‘lower’ attine *Apterostigma pilosum* (a coral fungus ant, representing the most basal of the attines). We also examined the behavioral activity elicited by the most abundant components of the alarm pheromones for the two *Acromyrmex* species, and compared this with the behavioral responses of the two *Atta* species to their most behaviorally active components. Finally, we examined how caste and age of *Acromyrmex* workers affect their responsiveness to alarm pheromone. We did this using a precise, individual-level assay based on the mandible opening response (Guerrieri and D’Ettorre 2008), a response which is stimulated in ants by alarm pheromones regardless of whether ants exhibit a panic or aggressive response (Hölldobler and Wilson 1990; Hughes et al. 2001b; Norman et al. 2014).

## Methods and Materials

### Chemical Composition of Alarm Pheromone in Attine Ants

Workers were collected from colonies of *Ap. pilosum*, *S. amabilis*, *T. cornetzi*, *Ac. echinatior*, *Ac. octospinosus*, *At. cephalotes* and *At. colombica* that had been collected from Gamboa, Panama in May 2013 and maintained in the laboratory for 6–18 months. We collected 20 workers from each of 2–4 colonies of the monomorphic *Ap. pilosum* (3 colonies), *T. cornetzi* (2 colonies) and *S. amabilis* (4 colonies). For the polymorphic leaf-cutting ants, we collected 20 small (<1.5 mm mm head width) and 20 large (2.0–3.0 mm head width) workers from 2 *to* 4 colonies of *Ac. echinatior* (4 colonies), *Ac. octospinosus* (3 colonies), *At. colombica* (2 colonies) and *At. cephalotes* (2 colonies). Ants were immediately cooled on ice after collection, and their heads removed and placed into 100 μl hexane (97% pure) containing 10 ng/ant head of an internal standard (5-methyl-3-heptanone 97%, Sigma-Aldrich, St Louis, MO). Heads were crushed thoroughly with a clean glass rod, vortexed for 60 s, placed in a sonic bath for 60 s, spun down for 60 s, filtered through a glass Pasteur pipette with a plug of clean glass wool, and the filtered extract stored at −20 °C until analysis. Crushed heads were used because previous work showed no difference in the composition of major volatiles present in mandibular glands and that released by crushed heads (Blum et al. 1968; Do Nascimento et al. 1993; Francelino et al. 2008; Hernández et al. 2002; Moser et al. 1968; Murakami et al. 2000; VN, FD and WOHH, unpubl. Data)

The sample extracts were analyzed by gas chromatography/mass spectrometry (GC/MS), using a Perkin Elmer Autosystem XL GC and TurboMass MS. The column used was a Supelco SLB-5 ms, 30 m × 0.25 mm ID × 0.25 μm film thickness (Sigma-Aldrich). The temperature program was 40 °C (held for 3 min), then 10 °C.min^−1^ to 75 °C, 20 °C.min^−1^ to 300 °C, and held for 5 min. Samples were injected using a splitless injector with a purge time of 1.0 min; the inlet temperature was 250 °C and injection volume 0.3 μl. The carrier gas was helium at 1.3 ml.min^−1^. The instrument was operated in full scan mode with range *m/z* 20–650. Transfer line and MS source temperatures were 300 °C and 250 °C, respectively. Compounds were identified by searching a database (NIST MSSearch 2.0 g) for matching mass spectra then confirmed by comparing retention times and mass spectra with authentic standards.

### Experiment 1: Behavioral Activity of Compounds in Leaf-Cutting Ants

In order to determine the behaviorally active components of the alarm pheromone, the mandible-opening response (MOR) assay was used (Guerrieri and D’Ettorre 2008; Norman et al. 2014). This individual-level assay is ideal because individual ants can be isolated and their threat response toward specific chemical stimuli under controlled conditions can be easily ascertained by whether or not the focal ant gapes its mandibles after exposure to the stimulus (indicating recognition of a threat, and therefore capturing both panic and aggression responses) (Hölldobler and Wilson 1990; Hughes et al. 2001b; Norman et al. 2014).

To carry out the MOR assay, ants were chilled on ice and then harnessed in 0.2 ml pipette tips (Starlab, Bucks, UK), cut at the apex and through which the head of the ant was passed, and secured with a thin strip of masking tape. Ants were left for 2 h in the harness to acclimatize before being assayed, and for at least 30 min between chemical stimuli to avoid habituation. The order of presentation of the stimuli to individuals was randomized. In order to test which of the chemicals identified in the alarm pheromone extract were active, compounds identified as consistently present in the extracts of either *Atta* or *Acromymex* species were tested on their respective species using the MOR. Neat compounds were used for this assay, as preliminary data showed these elicited the highest levels of response (V. C. N., unpublished data). We compared the responses of 30 large workers (2.0–2.5 mm head width) and 30 small workers (1.0–1.5 mm head width) for each species, sampled equally from five colonies of *Ac. echinatior*, *Ac. octospinosus* and *At. colombica*, or from the single available colony of *At. cephalotes*. To simulate the volatile emission of alarm pheromone by ants, a 20 μl drop of neat compound was placed on filter paper (10 × 20 mm) 10 mm from the antennae of the focal ant, and the response of the ant recorded. A 20 μl drop of water applied in the same way was used as a negative control, and the crushed head of a freshly freeze-killed nestmate used as a positive control.

### Experiment 2: the Effect of Caste and Age on Alarm Response in Acromyrmex

To determine if the caste or age of *Ac. echinatior* and *Ac. octospinsus* leaf-cutting ant workers affected their response to alarm pheromone, we again utilized the MOR assay. To examine the effect of caste, we compared the responses of 30 small workers, 30 medium workers, and 30 large workers (1.25 ± 0.03, 1.65 ± 0.02, and 2.09 ± 0.03 mm head widths, respectively, sampled equally from five colonies of each species). All ants were of medium cuticular coloration, indicating similar age (Armitage and Boomsma 2010). This was confirmed by quantifying the cuticular coloration of a subset of 30 ants/caste from a dorsal photograph taken with a Canon EOS 350d dSLR camera and Canon EF 100 mm f/2.8 Macro lens under constant lighting. Images were imported into ImageJ software and converted to grayscale, giving a reading of 0 (pure black) to 256 (pure white). Cuticular color was quantified using the mean value of the middle third of the femur of one of the rear legs, as in Armitage and Boomsma (2010). To examine the effect of age on the threat response, ants of similar size (1.2–1.8-mm head width) and of three different putative age classes (young, medium and old) were selected based on their cuticular coloration. Six ants of each age class were chosen from each of the five colonies for each species, giving 90 ants in total per species. To confirm age class, each ant was photographed and cuticular coloration quantified as described above. Mean ± s.e. color for each of the three putative age categories was: young 93.4 ± 1.4, medium 70.5 ± 0.75 and old 57.6 ± 0.93 (Fig. S2). In both the caste and age experiments, we tested the responses of ants to the most behaviorally active compound (3-octanol for *Ac. echinatior* and 3-octanone for *Ac. octospinosus*) at a dosage corresponding to the amount found in a single large worker ant head (25 ng for *Ac. echinatior*, 135 ng for *Ac. octospinosus*). Compounds were dissolved in hexane to 1 ng/μl, placed on filter paper, left for 15 s to allow the solvent to evaporate, and the filter paper (10 × 20 mm) placed within 10 mm of the antennae of the focal ant. Worker’s responses to these ‘head-realistic’ doses were compared to a solvent negative control (prepared in the same way as the head-realistic doses, but with an equivalent volume of hexane only), and a crushed head of a freshly freeze-killed nestmate as a positive control.

### Experiment 3: Colony-Level Assay

To confirm that the MOR assay was appropriate for detecting alarm responses, we also carried out colony-level assays using *Ac. echinatior* and *Ac. octospinosus* to confirm, in a more realistic colony set-up, that the ants exhibited alarm behaviors in response to the compounds and that individuals showed mandible gaping when alarmed. For each of the compounds found consistently in the extracts of each species, 50 mm filter paper discs containing the equivalent amount found in one crushed ant’s head were used as alarm stimuli. Once applied to the disc, the solvent was left to evaporate for 15 s before the disc was placed at least 50 mm away from the nest entrance. Each assay was filmed for 1 min with snapshot behaviors recorded at 10 s intervals during this period. The number of ants gaping the mandibles was recorded at each 10 s, as well as the number of ants present on the filter paper to quantify attraction or arrestment. This was carried out for four *Ac. octospinosus* colonies and three *Ac. echinatior* colonies. A crushed head of a freshly freeze-killed nestmate was used as a positive control.

### Experiment 4: Confirmation of Behavioral Responses to Volatiles Released by Ants

Finally, to confirm that focal ants were reacting to volatiles released by alarmed ants, and not just to other compounds in crushed whole heads, we carried out a further experiment utilizing the MOR assay. Focal ants were exposed to five stimuli in random order: 1) a live alarmed nestmate (with gaping mandibles indicating that the stimulus ant was alarmed and releasing alarm pheromone) held 10 mm away from the head of the focal ant, 2) a dead nestmate, which could not be releasing alarm pheromone, as a control, 3) eight live nestmates, in a sealed pot (25 mm diam., 40 mm height), that had been alarmed for 20 s, with the pot then placed 10 mm from the focal ant and opened, 4) the same treatment but under red light, to ensure focal ants were not reacting to the sight of alarmed ants, and 5) an empty pot as a control. These assays were carried out for 40 *Ac. echinatior* individuals (sampled evenly from four colonies) and 50 *Ac. octospinosus* individuals (sampled evenly from five colonies).

### Statistical Analysis

The programme PRIMER 6, version 6.1.13, was used with the permutational multivariate analysis of variance (*PERMANOVA*) version 1.0.3 for multivariate analysis of percentage composition to determine differences among species of the compounds present in the alarm pheromone. This analysis, a non-parametric *MANOVA,* has the advantage of being free from assumptions on data distribution (Anderson et al. 2008). All multivariate analyses were carried out using 9999 permutations on a resemblance matrix using Euclidian distance estimates. We used a one-factor *PERMANOVA* design, with species identity as a fixed factor. All peaks identified as present in the alarm pheromone (41 in total over the 7 species) were included in the analysis. A further *PERMANOVA* model was constructed to calculate post-hoc pairwise comparisons between species groups. We also carried out a one-way similarity of percentage analysis (*SIMPER*), to analyze further qualitative differences among species in alarm pheromone composition. This calculates the contributions of specific chemicals to the separation of species by the chemical composition in a non-metric multidimensional scaling analysis (*MDS*). A canonical analysis of principal components (*CAP*) was also carried out in order to predict group membership and help confirm the effectiveness of the functions in discriminating among the groups.

All behavioral data were analyzed using generalized linear mixed models (GLMM), which included colony-of-origin as a random factor, except for *At. Cephalotes*, for which only one colony was used and the data, therefore, analyzed with a generalized linear model (GLM). For colony-level assays, time point nested within colony was used as a random factor. For colony-level assays, number of ants and number of MORs were analyzed using a Poisson distribution and a log-link function. All individual-level MOR experiments were compared using binomial distributions and a logit-link function, with stimuli in the first experiment, and stimuli and caste or age in the second experiment, as factors. Non-significant interaction terms were removed stepwise in all cases to obtain the minimum adequate models. Sequential Bonferonni corrections were used to adjust for multiple comparisons during pairwise post-hoc testing. All behavioral analyses were performed in SPSS (v.20 SPSS Inc., Chicago, IL, USA).

## Results

### Chemical Composition

**Fig. 1:**
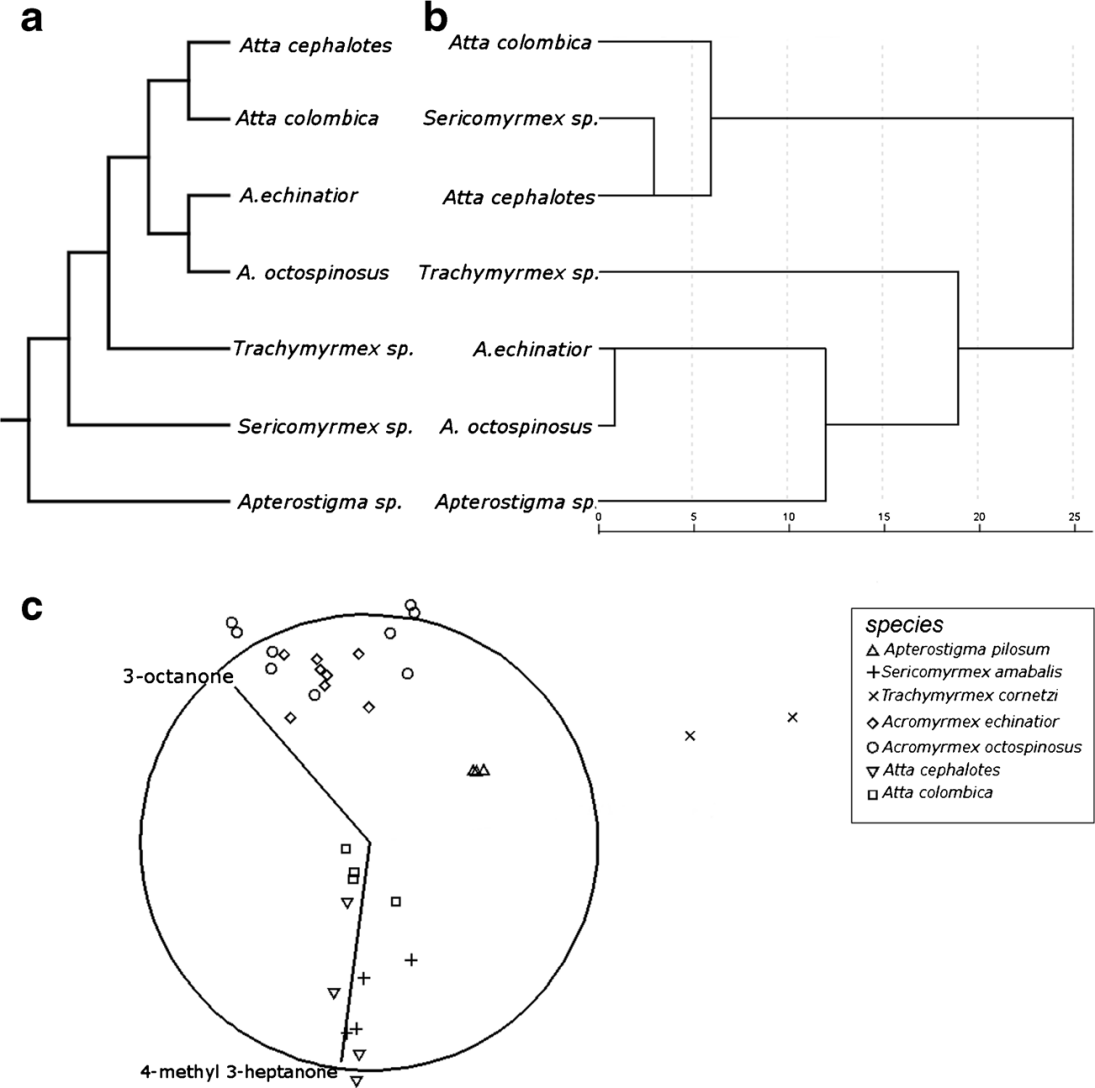
Composition of the alarm pheromone of seven attine fungus-growing ants species. (**a**) Phylogeny of the seven attine species (adapted from Schultz and Brady 2008) and (**b**) dendrogram showing similarity in the composition of alarm pheromone composition, calculated using Euclidian distances, with shorter distances between chemical profiles indicating greater similarity. (**c**) Multi-directional scaling (MDS) plot, showing the similarity of alarm pheromone composition for the seven attine species: *Apterostigma pilosum* (open upright triangles), *Trachymyrmex cornetzi* (rotated crosses), *Sericomyrmex amabalis* (crosses), *Acromyrmex echinatior* (open diamonds), *Acromyrmex octospinosus* (open circles), *Atta cephalotes* (open upturned triangles) and *Atta colombica* (open squares) calculated using Euclidian distances. Each symbol is from a sample of 20 workers from one colony, with shorter distances between symbols indicating greater similarity in pheromone composition. Straight lines indicate the main axis of differentiation between the samples and the main chemicals causing this grouping

Alarm pheromone composition differed among the seven species tested (F_6,27_ = 20.3; *P* < 0.001). The species differed in their compositions, except for *Ac. octopsinosus* and *Ac. echinatior* (*P* = 0.521), and also *At.cephalotes* and *S. amabilis* (*P* = 0.604). There was little congruence across genera between their phylogeny and the chemical similarity of their alarm pheromone (Fig. 1a). This was supported by MDS analysis, which separated the species into three clusters, with the *Acromyrmex* species clustering together mostly differentiated from other attines by the presence of 3-octanone (Fig. 1b). Interestingly, *S. amabilis* clustered with the two *Atta* species, differentiated from the other attines by the presence of 4-methyl-3-heptanone. *Trachymyrmex cornetzi* and *Ap. pilosum* made up the third cluster and were both mostly distinguished from the other two clusters by the almost complete lack of either 4-methyl-3-heptanone or 3-octanone. In total, 29 compounds of high volatility occurred consistently in one or more of the species. Of these, 24 compounds could be identified with a high degree of confidence, including all of the most abundant peaks (Table 1). The CAP analysis correctly identified species in 91% of the 34 samples. Misclassification of species only occurred in 3 out of the 7 species, with 25% of samples being misclassified for *At. cephalotes* (1/4 samples), *At. colombica* (1/4) and *S. amabilis* (1/4), in all cases as other species within this cluster.

**Table 1:** Volatile compounds found in the mandibular gland pheromone of seven attine fungus-growing ant species

				Volatiles (% mean ± s.e)							
Compound	*Apterostigma pilosum*	*Sericomyrmex amabalis*	*Trachymyrmex cornetzi*	*Acromyrmex octospinosus*		*Acromyrmex echinatior*		*Atta colombica*		*Atta cephalotes*	
				LW	SW	LW	SW	LW	SW	LW	SW
2-ethyl-1-hexanol	0	4.39 ± 1.56	0	4.23 ± 3.33	2.05	5.98 ± 2.02	1.48 ± 3.99	19.4 ± 2.9	17.6 ± 8.8	19.81	10.8 ± 10.8
3-octanol	0	0	4.1 ± 0.9	4 ± 2.23	2.9	6.22 ± 2.39	5.0 ± 2.71	0	14.2 ± 7.1	0	0
3-octanone	2.04 ± 0.286	0	0	**38.5 ± 13.5**	**41.12**	**40.6 ± 1.06**	**42.0 ± 1.70**	2.47 ± 1.8	16.2 ± 8.1	0	0.933 ± 0.93
4-methyl-3-heptanone	0	**70.7 ± 5.34**	0	0	0	0	0	**41.7 ± 2.3**	**41.6 ± 2.9**	**69.7**	**72.0 ± 14.6**
2-nonanone	0	0	0	3.33 ± 2.06	5.18	6.38 ± 2.46	7.4 ± 2.86	0	0	0	0
2-undecanone	0	0	0	28.9 ± 8.96	36.97	24 ± 1.31	33.5 ± 1.48	0	1.61 ± 0.8	0	0
nonanal	0	0	0	0	0	0.725 ± 3.32	0	0	0	0	0
nonane	0	0	0	4.93 ± 1.09	1.367	13.8 ± 1.21	4.0 ± 2.41	6.7 ± 0.6	2.5 ± 0.28	5.32	1.02 ± 1.02
4-methyl-3-heptanol	0	8.4 ± 7.36	1.7 ± 1.7	0	0	0	0	4.8 ± 0.2	9.97 ± 6.7	0	5.04 ± 2.96
octanal	0	1.31 ± 0.736	0	0.567 ± 0.57	0	0	0	0	0	0	0
2-heptanone	0	0	0	0	0	0	0	0	3.50 ± 1.6	0	0
4-methyl-3-hexanol	0	0.695 ± 0.695	0	0	0	0	0	0	0	0	0
4-methyl-3-hexanone	0	5.49 ± 0.879	0	0	0	0	0	0	0	0	0
1-octen-3-ol	0	0	0.6 ± 0.6	0	0	0	0	0	0	0	0
3-methyl-2-hexene	0	0	**84.1 ± 9.56**	0	0	0	0	0	0	0	0
3-heptanone	0	2.67 ± 0.32	0	0	0	0	0	0	0	0	0
3,5-dimethyl-2-octanone	14.1 ± 0.83	0	0	0	0	0	0	0	0	0	0
2-methyl-2-heptenal	6.03 ± 0.62	0	0	0	0	0	0	0	0	0	0
2-Dodecenal	**22.4 ± 2.12**	0	0	0	0	0	0	0	0	0	0
citral variant	0	0	0	3.23 ± 2.04	0.65	0	0	0	0	0	0
1-octen-3-one	3.34 ± 0.23	0	0	0	0	0	0	0	0	0	0
3,4-dimethyl-2-hexanone	5.23 ± 0.60	0	0	0	0	0	0	0	0	0	0
8-methyl-1-undecene	8.53 ± 0.70	0	0	0	0	0	0	0	0	0	0
3,5-dimethyl-octanone	6.27 ± 0.34	0	0	0	0	0	0	0	0	0	0
U9.1	0	1.3 ± 1.10	0	0	0	0	0	0	0	0	0
U9.9	19.2 ± 0.38	4.81 ± 1.06	0	0	0	0	0	0	0	0	0
U9.6	0	0	9.3 ± 7.9	0	0	0	0	0	0	0	0
U10.9	0	0	0	2.67 ± 2.67	1.28	0	0	0	0	0	0
U14.4	0	0.736 ± 0.65	0	6.93 ± 3.76	3.48	0	1.74 ± 2.59	15.4 ± 1.3	0	0	2.00 ± 2.00
Total amount (pg/μl/ant head)	26.2 ± 0.87	0.24 ± 0.13	4.08 ± 0.57	0.37 ± 0.037	0.20 ± 0.02	0.44 ± 0.03	0.024 ± 0.0053	0.2 ± 0.07	0.05 ± 0.01	0.184	0.21 ± 0.07

The most abundant compound for each species is highlighted in bold and the most behaviorally active is underlined

U signifies an unknown compound

### Experiment 1: Behavioral Activity of Compounds in Leaf-Cutting Ants

**Fig. 2:**
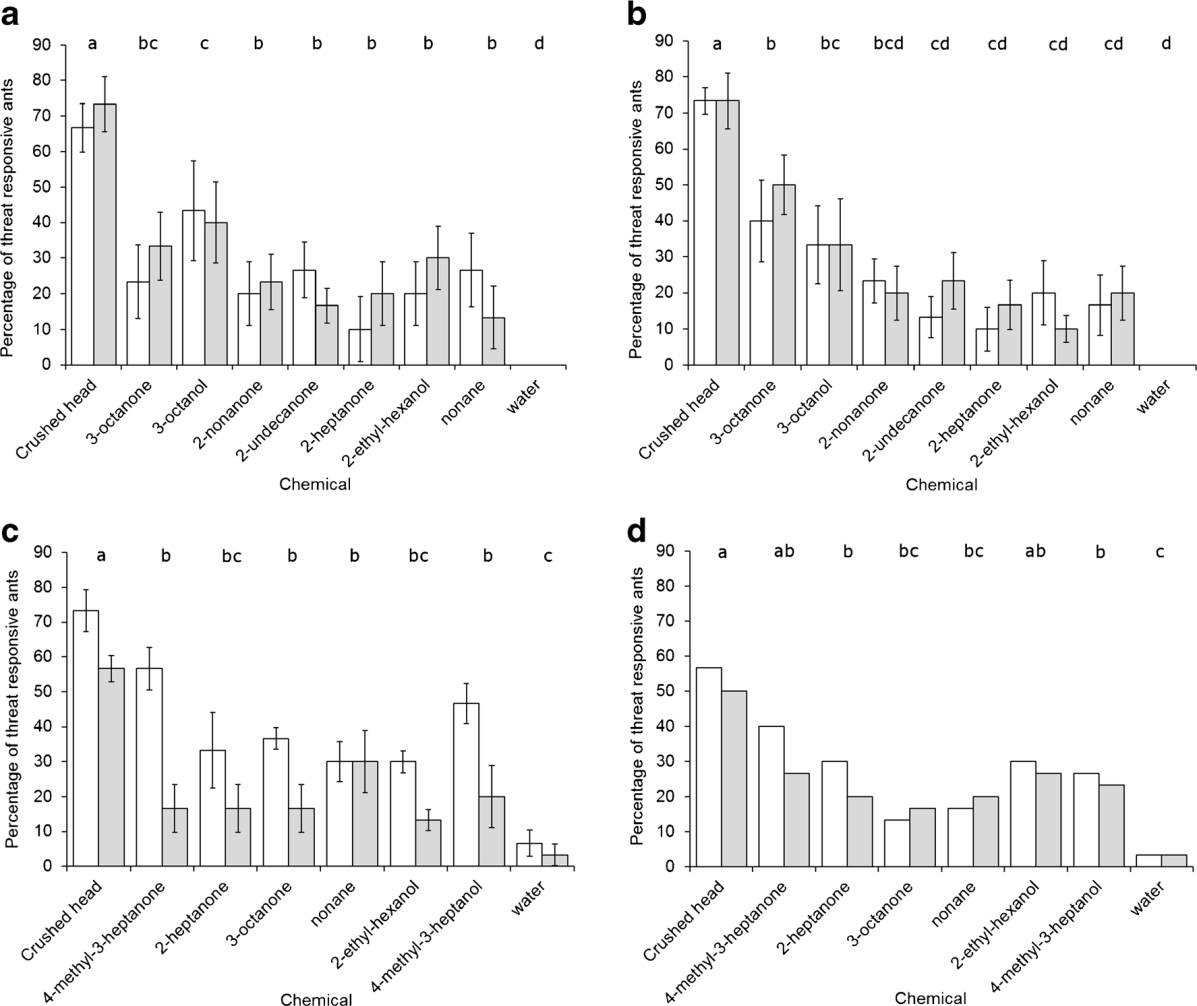
Behavioral responses of four species of leaf-cutting ants to the most abundant compounds in their alarm pheromones. Mean ± s.e percentages of ants showing a positive mandible opening response to compounds found in crushed ant heads for 30 small workers (*white bars*) and 30 large workers (*grey bars*), sampled evenly among colonies for (**a**) *Acromyrmex echinatior* (5 colonies), (**b**) *Acromyrmex octospinosus* (5 colonies), (**c**) *Atta colombica* (5 colonies) and (**d**) *Atta cephalotes* (1 colony). Different letters above columns indicate treatments that differed from each other at *P* < 0.05 in pairwise comparisons

All four species of leaf-cutting ants showed differences in their MOR behavior to the different compounds tested (F_8,530_ = 7.99, *P* < 0.001, F_8,530_ = 8.93, *P* < 0.001, F_7,471_ = 6.62, *P* < 0.001, χ^2^_8_ = 51.8, *P* < 0.001 for *Ac. echinatior*, *Ac. octospinosus*, *At. colombica* and *At. cephalotes*, respectively; Fig. 2). For all species, crushed heads caused more positive MORs than any of the single compounds tested, and there were minimal responses to the water negative control. The most behaviorally active compound for both *At. cephalotes* and *At. colombica* was 4-methyl-3-heptanone (Fig. 2). The most behaviorally active compound for *Ac. echinatior* was 3-octanol, whereas for *Ac. octospinosus* it was 3-octanone (Fig. 2). There was no difference between large and small workers in their responses to neat compounds for *Ac. echinatior*, *Ac. octospinosus* and *At. cephalotes* (F_1,530_ = 0.204, *P* = 0.653; F_1,530_ = 0.315, *P* = 0.989, χ^2^_1_ = 0.995, *P* = 0.319, respectively), whereas for *At. Colombica*, small workers were more threat responsive than large workers (F_1,471_ = 19.1, *P* < 0.001; Fig. 2c).

### Experiment 2: the Effect of Caste and Age on Alarm Response in Acromyrmex

**Fig. 3:**
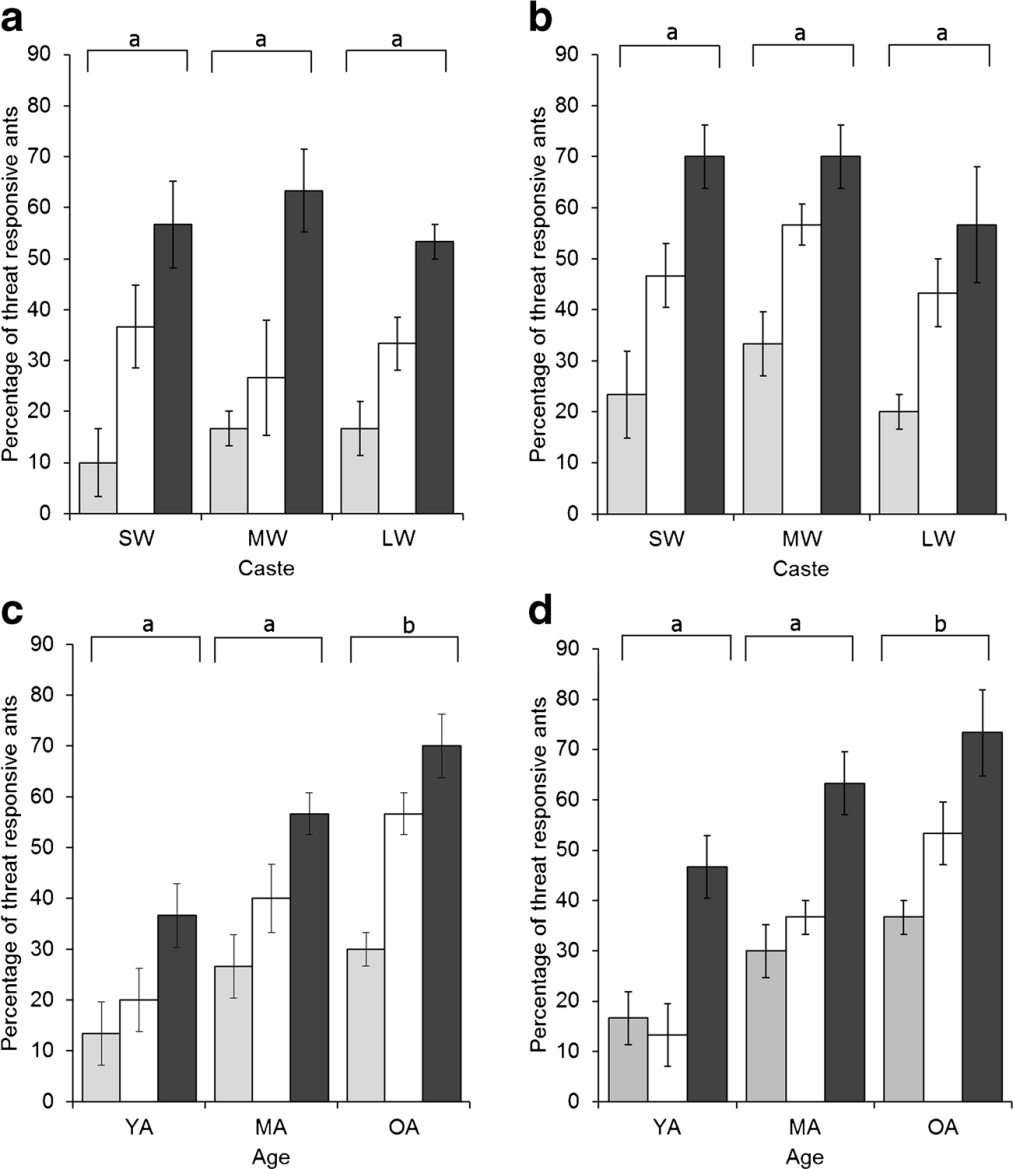
The effect of caste and age on the behavioral responses of two *Acromyrmex* leaf-cutting ant species to head-realistic concentrations of the most behaviorally active compounds in their alarm pheromone. Mean ± s.e percentages of ants showing a positive mandible opening response (MOR) to solvent negative control (light grey bars), head realistic doses of the most behaviorally active compound (white bars) and crushed nestmate head as a positive control (dark grey bars). Data are for small workers (SW), medium workers (MW) and large workers (LW) of (**a**) *Acromyrmex echinatior*, and (**b**) *Acromyrmex octospinosus*, and for young (Y), medium (M) and old (O) workers of (**c**) *Acromyrmex echinatior* and (**d**) *Acromyrmex octospinosus*. Different letters above sets of columns indicate differences among castes or ages of ants at *P* < 0.05 in pairwise comparisons

Ants in all assays responded most strongly to crushed head positive controls, followed by head-realistic doses of the most behaviorally active compound, and showed the least response to the negative control (Fig. 3). For both *Ac. echinatior* and *Ac. Octospinosus*, there was no effect of caste on the proportion of ants showing a MOR (F_2,265_ = 0.019, *P* = 0.981, F_2,265_ = 1.81, *P* = 0.166, respectively; Fig. 3a,b). However, there was an effect of putative age class on the MOR for both *Ac. echinatior* and *Ac. octospinosus* (F_2,265_ = 17.0, *P* < 0.001, F_2,265_ = 14.0, *P* < 0.001, respectively). Older workers were more threat responsive than younger workers, for all stimuli and for both species (Fig. 3c,d). All color classes, indicating the putative age classes, were different from one another in cuticular color (F_2,174_ = 347.4, *P* < 0.001; Fig. S2).

### Experiment 3: Colony-Level Assay

**Fig. 4:**
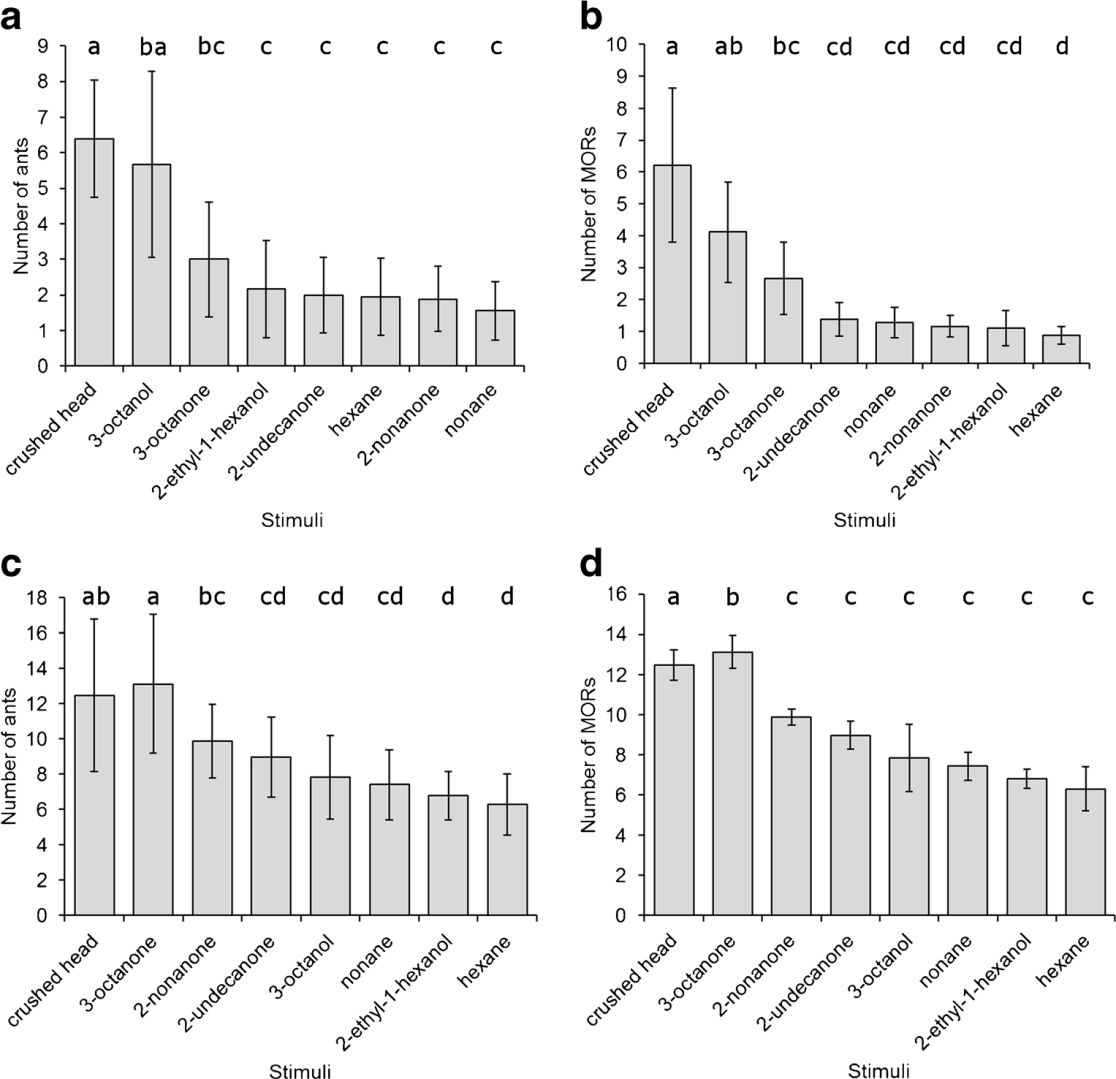
Behavioral responses of *Acromyrmex octospinosus* (**a**, **b**) and *Acromyrmex echinatior* (**c**, **d**) leaf-cutting ant colonies to the most abundant compounds in their alarm pheromones. Mean ± s.e. number of ants within 50 mm of the alarm stimulus (**a**, **c**) and number of ants exhibiting a mandible opening response (MOR) (**b**, **d**). Different letters above columns indicate treatments that differed from each other at *P* < 0.05 in pairwise comparisons

There were differences among treatments in the numbers of ants within 50 mm of the stimulus (F_7,136_ = 18.6, *P* < 0.001, F_7,184_ = 16.8, *P* < 0.001 for *Ac. echinatior* and *Ac. octospinosus*, respectively). The numbers of ants attracted or arrested near the stimulus were higher in response to crushed heads and either 3-octanol (for *Ac. octospinosus*) or 3-octanone (for *Ac. echinatior*), than in response to the other compounds or the control (Fig. 4a,c). The treatments similarly differed in the numbers of ants exhibiting gaping mandibles following presentation of the stimuli (F_7,136_ = 23.0, *P* < 0.001, F_7,184_ = 54.2, *P* < 0.001 for *Ac. echinatior* and *Ac. octospinosus*, respectively), with more ants showing gaping mandibles in response to crushed heads and 3-octanol, or 3-octanone (depending on species), than to the other compounds or control (Fig. 4).

### Experiment 4: Confirmation of Behavioral Responses to Volatiles Released by Ants

For both *Ac. octospinosus* and *Ac. echinatior*, there were differences among treatments (F_4,195_ = 4.147, *P* = 0.003, F_4,232_ = 9.361, *P* < 0.001 for *Ac. echinatior* and *Ac. octospinosus*, respectively) in the numbers of ants exhibiting MOR. Ants showed the highest frequencies of MOR in response to alarmed nestmates with more gaping of mandibles in response to 1 or 8 nestmates than to either a dead nestmate or an empty pot. Identical levels of alarm were produced under red light, simulating darkness for focal ants (Fig. S1).

## Discussion

Contrary to the prediction of little interspecific variation in alarm pheromone composition, we found composition differed across the attine clade, with 4-methyl-3-heptanone being the most abundant compound in *S. amabalis* and *Atta spp.*, but *Acromyrmex spp*. utilize different behaviorally active compounds. Furthermore, in contrast to previous studies on *Atta*, we found that *Acromyrmex* workers show no caste differences in behavioral response to alarm pheromone, but do exhibit behavioral differences based on putative age class. As in previous studies (e.g., Cammaerts et al. 1983; Moser et al. 1968), no individual compound elicited the same level of behavioral response as the natural pheromone, with the most active compound for each species eliciting a response 60–90% of that of the natural pheromone, suggesting multiple compounds are needed to elicit full alarm responses.

Although there is a general prediction of a lack of interspecific specificity in alarm pheromones and cues across the animal kingdom (Blum 1969; Commens and Mathis 1999; Laforsch et al. 2006; Vander Meer and Alonso 1998), it might be expected that taxa with larger, more complex societies might evolve ‘better’, more informationally rich, mixtures of alarm pheromone (Hölldobler 1995; Wilson and Regnier 1971). This could occur because of the greater quantity of resources they have to defend, and the greater availability and specialization of workers they have with which to respond (Hölldobler and Wilson 1990). The dramatic increase in colony size and complexity across the attines, from tens of monomorphic workers in basal taxa to millions of polymorphic workers in *Atta* (Weber 1972) suggest potential changes in alarm pheromone composition. However, our results provided no support for either factor affecting pheromone composition. The composition of the mandibular gland pheromone differed little in complexity across the attines and, while the main compound in both *Atta* leaf-cutting species was 4-methyl-3-heptanone in keeping with previous studies of *Atta* (Francelino et al. 2006; Hernandez et al. 1999; Hughes et al. 2001a; Moser et al. 1968), this compound was also the most abundant in *S. amabalis*. Furthermore, the other leaf-cutting ant genus *Acromyrmex* lacked this compound and appears to use 3-octanone or 3-octanol instead. *Trachymyrmex cornetzi* and *Ap. pilosum* had unique main constituents compared to the other attines and, although we found very small traces of 4-methyl-3-heptanone, 3-octanol and 3-octanone, previously found in *T. septentrionalis* and *T. seminole* (Crewe and Blum 1972), the most abundant peaks for these two attines were 3-methyl-2-hexene and 2-dodecenal. One potential explanation for these interspecific differences may be that the components of the mandibular gland secretions serve other purposes, such as being antimicrobial (North et al. 1997). Alternatively, they may have evolved to allow some interspecific recognition, as *Acromyrmex* and *Atta* are sympatric competitors, or may be epiphenomenal to differences in diet. The case here is particularly interesting within the genus *Acromyrmex*, as compounds are found in the same abundance between these sympatric species but have different behavioral activity (3-octanone and 3-ocatanol being most behaviorally active, respectively, for *Ac*. *octospinosus* and *Ac*. *echinatior*). Regardless of the reason, it seems that there can be remarkable interspecific variability in the composition of alarm pheromone even between closely related taxa, and further investigation of both the proximate and ultimate explanations for this would be worthwhile.

There was polyethism in the propensity of *Acromyrmex* workers to respond to alarm pheromone. For both *Ac. echinatior*, and *Ac. octospinosus*, there was no difference in MORs (indicating a focal ant has perceived a threat) among morphological castes (small, medium and large age-matched workers), but there were differences among size-matched young, medium and old putative age classes. Differences in alarm behavior between morphological castes are known in *Atta* leaf-cutting ants, both in the production of alarm pheromone and behavioral responses to it (Francelino et al. 2006; Hernandez et al. 1999; Hughes et al. 2001a). Smaller workers in *Atta* respond in higher frequency to conspecific intruders and are more responsive to alarm pheromone (Hughes and Goulson 2001; Wilson 1980; Whitehouse and Jaffe 1996), consistent with our behavioral data. *Acromyrmex* leaf-cutting ants share many similarities with *Atta*, including worker polymorphism that affects division of labor, so the lack of an effect of size on response to alarm pheromone in *Acromyrmex* is somewhat surprising. Previous MOR studies with *Acromyrmex* have similarly found no difference among physical castes in threat responsiveness (Norman et al. 2014). It may be that the lack of morphological caste differences with respect to nest defence is indicative of *Acromyrmex* having a simpler division of labour than *Atta*, or maybe because individuals express a far less extreme alarm response.

Age of workers, even more than morphological caste, is an important and widespread basis for division of labor in social insects, including in *Acromyrmex* leaf-cutting ants (Camargo et al. 2007; Seeley 1982; Waddington and Hughes 2010). In both *Acromyrmex* species tested here, putative age class affected the propensity of an individual to show an MOR toward chemical stimuli (with older ants responding more frequently). Older *Ac. echinatior* individuals are also more threat responsive than young individuals to non-nestmates, and similar relationships with older workers being more aggressive or threat responsive have been reported in many other social insects (Norman et al. 2014; Robinson 1987; Santoro et al. 2015; Waddington et al. 2010; van Wilgenburg et al. 2010). This relationship is likely to be beneficial in insect societies because age polyethism results in older workers spending more time outside of the nest, where they are more likely to encounter threats (Moore et al. 1987). It may be that younger ants are less threat responsive because their longer potential lifespan makes them more valuable to the colony than the older workers, which spend more time outside the nest carrying out behaviors more relevant to alarm cues (Camargo et al. 2007).

The surprising level of interspecific differences seen across the attines in this study highlights the need for detailed, comparative work on the alarm pheromones of fungus-growing ants in particular as well as social insects in general. It will be very interesting to discover whether the logical premise of low interspecific variation in alarm pheromone composition generally holds true and, if not, then why not? The simplicity of alarm pheromones and the interspecific variation in composition that they show in this system offer much potential for studying and understanding the proximate and ultimate basis for the production of pheromones.

## Electronic supplementary material

Fig. S1 (DOCX 67 kb)

Fig. S2 (DOCX 35 kb)

ESM (XLSX 125 kb)
