# Supplementary material for: Alarm Pheromone Composition and Behavioral Activity in Fungus-Growing Ants
Source: J Chem Ecol. 2017 Feb 28;43(3):225–35. doi: 10.1007/s10886-017-0821-4 (PMC5371636; doi:10.1007/s10886-017-0821-4)
Supplement: Supplementary file 1 — (DOCX 67 kb) [file 10886_2017_821_MOESM1_ESM.docx]

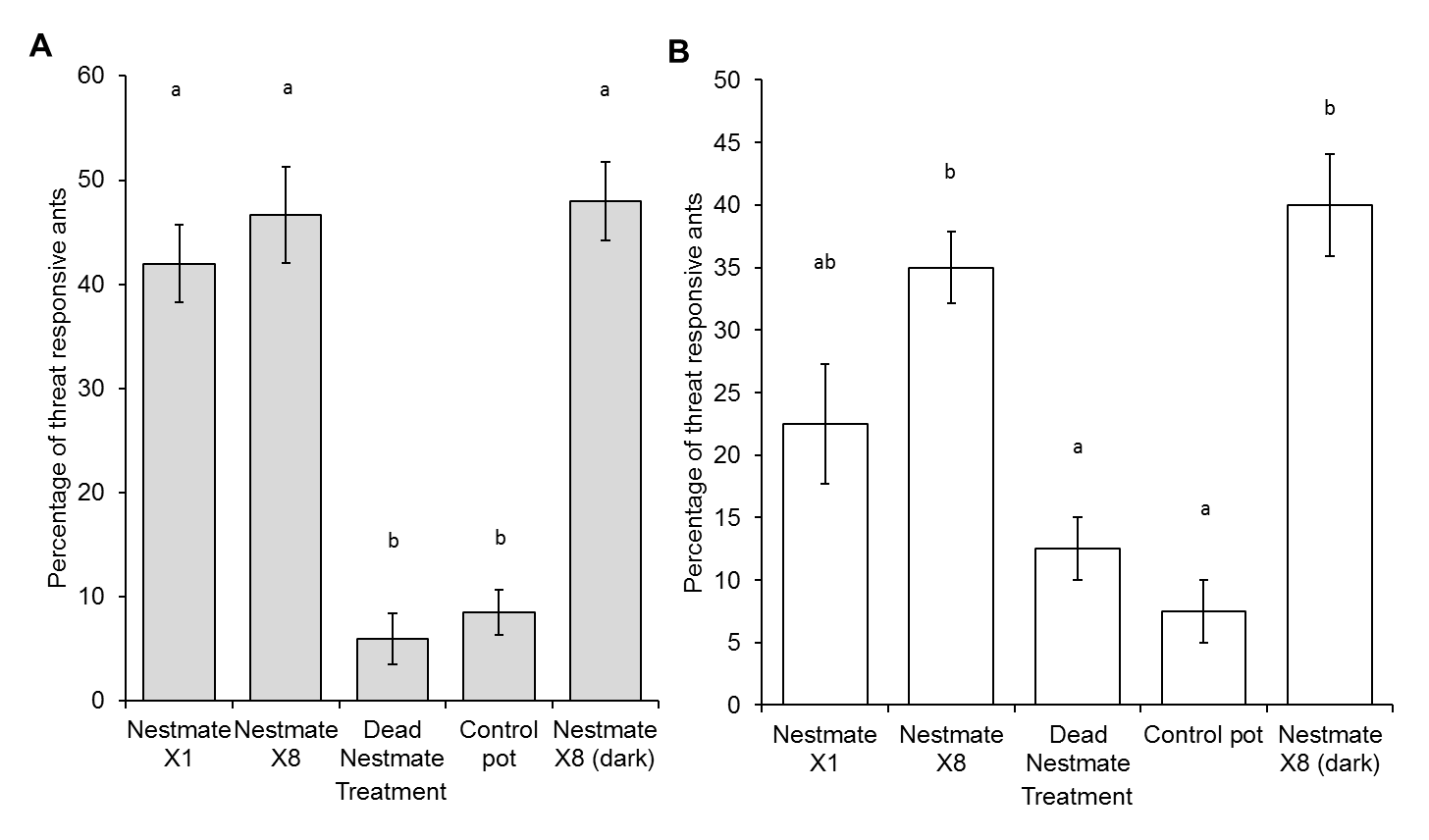


**Fig. S1** The behavioural responses of both a) *Acromyrmex octospinosus* and b) *Acromyrmex echinatior*  to live alarming nestmates. Mean ± s.e. percentage of positive mandible opening responses (MORs) to live ants emitting alarm pheromone for either 1 nestmate, 8 nestmates, their respective controls as well as one of the treatments under red light to confirm focal ants were responding to the volatiles emitted by alarming ants rather than the sight of alarming nestmates. Different letters above columns indicate treatments that differed significantly from one another at P < 0.05 in pairwise, post-hoc comparisons.
