# Supplementary material for: Alarm Pheromone Composition and Behavioral Activity in Fungus-Growing Ants
Source: J Chem Ecol. 2017 Feb 28;43(3):225–35. doi: 10.1007/s10886-017-0821-4 (PMC5371636; doi:10.1007/s10886-017-0821-4)
Supplement: Supplementary file 2 — (DOCX 35 kb) [file 10886_2017_821_MOESM2_ESM.docx]

**Fig. S2** The cuticular colouration of young, medium and old putative age cohorts. Mean ± s.e. cuticular colouration (on a 0-256 scale of pure black to pure white). Cuticular colouration was quantified using the mean value of the middle third of the femur of one of the rear legs as in Armitage and Boomsma (2010). All putative age classes are significantly different from one another (P<0.001) in pairwise, post-hoc comparisons.
